# Morroniside Improves Microvascular Functional Integrity of the Neurovascular Unit after Cerebral Ischemia

**DOI:** 10.1371/journal.pone.0101194

**Published:** 2014-06-30

**Authors:** Fang-Ling Sun, Wen Wang, Hua Cheng, Ying Wang, Lei Li, Jin-Long Xue, Xiao-feng Wang, Hou-Xi Ai, Li Zhang, Jing-dong Xu, Xiao-Min Wang, Xun-Ming Ji

**Affiliations:** 1 Department of Pharmacology, Xuanwu Hospital of Capital Medical University, Beijing, China; 2 Department of Physiology, School of Basic Medical Sciences, Capital Medical University, Beijing, China; 3 Department of Neurobiology, School of Basic Medical Sciences, Capital Medical University, Beijing, China; 4 Department of Neurosurgery, Xuanwu Hospital of Capital Medical University, Beijing, China; National University of Singapore, Singapore

## Abstract

Treating the vascular elements within the neurovascular unit is essential for protecting and repairing the brain after stroke. Acute injury on endothelial systems results in the disruption of blood-brain barrier (BBB), while post-ischemic angiogenesis plays an important role in delayed functional recovery. Here, we considered alterations in microvessel integrity to be targets for brain recovery, and tested the natural compound morroniside as a therapeutic approach to restore the vascular elements of injured tissue in a rat model of focal cerebral ischemia. Sprague-Dawley rats were subjected to middle cerebral artery occlusion (MCAO) model, and morroniside was then administered intragastrically once a day at doses of 30, 90, and 270 mg/kg. BBB integrity and associated factors were analyzed to identify cerebrovascular permeability 3 days after MCAO. The recruitment of endothelial progenitor cells (EPCs), the expression of angiogenic factors and the new vessel formation in the peri-infarct cortex of rats were examined 7 days after MCAO to identify the angiogenesis. We demonstrated that at 3 days post-ischemia, morroniside preserved neurovascular unit function by ameliorating BBB injury. By 7 days post-ischemia, morroniside amplified angiogenesis, in part by enhancing endothelial progenitor cell proliferation and expression of angiogenic factors. Morever, the increase in the amount of vWF^+^ vessels induced by ischemia could be extended to 28 days after administration of morroniside, indicating the crucial role of morroniside in angiogenesis during the chronic phase. Taken together, our findings suggested that morroniside might offer a novel therapeutic approach for promoting microvascular integrity recovery and provide a thoroughly new direction for stroke therapy.

## Introduction

Ischemic stroke is the most common form of stroke and is caused by the abrupt interruption of blood flow to the brain [1], [2]. Beyond thrombolysis for small subsets of cerebral ischemia patients, there are no therapies for stroke. The difficulty in drug development for stroke is perhaps best represented by the history of high-profile failures of a large number of neuroprotection clinical trials. However, the emerging concept of the “neurovascular unit” suggests that focusing on saving neurons alone is not sufficient. The relevance of dynamic interactions between cerebral endothelial cells, astrocytes, pericytes and neurons is emphasized for brain function and dysfunction after stroke.

During the early acute phase after stroke onset, besides basic cell death mechanisms, one of the most important facets of neurovascular damage is manifested as disruptions of blood–brain barrier (BBB) function [3], [4]. BBB breakdown is represented by the disruption of endothelial astrocyte-matrix interactions and leads to transmigration of inflammatory cells as well as toxic molecules into the brain parenchyma, which results in cerebral edema and hemorrhage [5]. During the delayed phase, a powerful array of endogenous mechanisms is recruited for neurovascular functional plasticity and recovery [6]. Angiogenesis is an important process in forming the new brain microvessels after cerebral ischemia, which can improve tissue microperfusion within the ischemic boundary region [7]. Even, a greater microvessel density in the ischemic border correlated with longer survival in stroke patients [8]. The angiogenic vessels may persist for more than 21 days following cerebral ischemia [9]. The growth of new blood vessels provides important source of neurotrophic support to newly generated neurons [10] and also serves as routes for neuroblasts to move into the recovering peri-infarct regions [11], [12]. Taken together, for vascular elements within the neurovascular unit, it is believed that the alterations in microvessel integrity might be targets for BBB function recovery, including cerebrovascular permeability during the early phase and the angiogenesis process during the late phase [13].

Morroniside is one of the most abundant iridoid glycosides extracted from *Cornus officinalis*. Our previous studies have demonstrated that besides the anti-oxidative and anti-apoptosis effects in vitro [14], [15], morroniside could reduce the infarct volume and promotes neurological functional recovery 3 days after focal ischemic stroke [16]. In the present study, we tested whether the natural compound morroniside could promote microvessel integrity repair and restore homeostasis in the neurovascular unit, thus improving recovery after stroke. This study may provide a potentially new strategy for stroke treatment.

## Methods and Materials

### Ethics statement

Animal protocols for this study were approved by the Animal Care and Use Committee of Xuanwu Hospital in Capital Medical University. The animals are fed and housed under a 12/12 h dark/light cycle and specific pathogen-free (SPF) conditions to ensured physical and psychological well-being. During the practice of research, we ensure the anaesthesia, surgery and post-operative care of the animals. Before MCAO model performed, the rats abstained from food overnight but were allowed free access to water. Anesthesia was induced and maintained with 3.5% enfluane in a mixture of 70% nitrous oxide and a balance of oxygen (Bickford veterinary anesthesia equipment model no. 61010, AM Bickford Inc., Wales Center, NY, USA). The rats were orally intubated, immobilized with intra-arterial pancuronium bromide (0.6 mg/kg) and mechanically ventilated (Rodent Ventilator Model 683, Harvard Apparatus Inc., Holliston, MA, USA). The right femoral artery was catheterized with a PE-50 tube for continuous blood pressure monitoring and periodic blood sampling for arterial gas levels, pH (248 pH/blood gas analyzer; Chiron Diagnostics Ltd, Halstead, UK) and plasma glucose (ACCU-CHEK Active GN 03173351, Roche, Mannheim, Germany) 15 minutes before and after the onset of MCA occlusion and 15 minutes before and after reperfusion. The mean arterial blood pressure was measured through an indwelling femoral arterial catheter connected to a pre-calibrated Statham pressure transducer and was recorded continuously. Serial measurements of arterial blood gas levels, pH and plasma glucose were made. Filament MCA occlusion was induced by an intraluminal filament model. Throughout the surgical procedure, the rectal temperature was maintained at 37.5°C with a circulating heating pad (Harvard Apparatus Ltd, Edenbridge, UK), and the left temporal muscle temperature was maintained at 37.5°C with a heating lamp. Next, a piece of nylon monofilament was inserted into the left internal carotid artery via an arteriotomy and lodged in the narrow proximal anterior cerebral artery, which blocked the MCA at its origin. After 30 min of ischemia, reperfusion was established by withdrawal of the filament. The rat’s body temperature was measured frequently through the rectum until it returned to normal levels. Animals from each group were placed on a heating pad under a heating lamp throughout the surgical procedure under anesthesia. When the cerebral and rectal temperature both returned to normal levels, the animals were allowed to regain consciousness and were placed under warm conditions for an additional 3 hours.

### Drug preparation

Morroniside was extracted from the sarcocarp of *C. officinalis* and purified as previously described [16]. *C. officinalis* was purchased from Tong Ren Tang Company, Beijing, China, and authenticated by Professor Wen Wang. The final purity of morroniside was determined to be 98.5% by high performance liquid chromatography (HPLC).

### Animals and the middle cerebral artery occlusion (MCAO) model

Male Sprague-Dawley (SD) rats weighing 260–280 g were purchased from Beijing Vitalriver Experimental Animal Co. (Beijing, China) and were housed under a 12/12 h dark/light cycle and specific pathogen-free (SPF) conditions. Before MCAO model was performed, the rats were fasted without water deprivation for 12 h. Focal ischemia was induced in enflurane-anesthetized rats for 30 minutes using the intraluminal vascular occlusion of the middle cerebral artery, as described previously [16], [17]. Blood pressure, blood gases, and blood glucose concentration were maintained in the normal range, and rectal temperature was maintained in the range of 36.5 to 37.5°C throughout the experiments. Sham operations were performed using the same anesthesia and surgical procedures but without insertion of the intraluminal filament.

The rats were randomly divided into five groups: a sham-operated group, a vehicle-treated ischemic model group, and morroniside-treated ischemic groups at dose of 30 mg/kg/day, 90 mg/kg/day, or 270 mg/kg/day. Morroniside was dissolved in normal saline and administered intragastrically once a day starting at 3 h after MCAO. The vehicle control groups of the ischemic and sham-operated rats received an equal volume of normal saline. Animal protocols for these studies were approved by the Animal Care and Use Committee of Xuanwu Hospital in Capital Medical University.

### Evans Blue (EB) staining

To analyze the cerebrovascular permeability after MCAO, the rats were injected with 1 ml of 2% EB (Ourchem, Beijing, China) 71 h after MCAO. After 1 h, the animals were perfused with PBS. The brains were removed and separated into hemispheres ipsilateral and contralateral to the MCAO. Each hemisphere was then homogenized in 2.5 ml of PBS and then in 2.5 ml of 60% trichloroacetic acid (Sigma-Aldrich, St. Louis, MO, USA). Next, the supernatants were collected after centrifuged for 30 min at 1000×*g* and detected at 610 nm. Briefly, the EB levels in each hemisphere were determined according to a standard curve and determined from the formula: (EB concentration×5 ml)/mg brain wet weight. The background EB levels in the non-ischemic hemisphere were subtracted from the ischemic ipsilateral hemisphere.

### Immunofluorescence and Immunohistochemistry analysis

Immunofluorescence staining was used to visualize von Willebrand factor (vWF)-positive vessels. Double-immunofluorescence was used for bromodeoxyuridine (BrdU) with laminin, and was also used for the localization of matrix metalloproteinase-9 (MMP-9), MMP-2, angiopoietin-1 (Ang-1), and Tie-2. BrdU (50 mg/kg; Sigma-Aldrich) was injected intraperitoneally twice daily for 4 consecutive days after MCAO. Next, the rats were perfused with 4% paraformaldehyde in PBS, and the brains were extracted and post-fixed overnight. Ice-cold sections (40 µm) were prepared using standard protocols. For BrdU staining, the sections were pretreated with 50% formamide/280 mM NaCl/30 mM sodium citrate at 65°C for 2 h, incubated in 2 M HCl at 37°C for 30 min, and rinsed in boric acid (0.1 M, pH 8.5) at room temperature for 10 min. After incubation with a blocking solution, all sections were incubated with primary antibodies for 48 h at 4°C. Primary antibodies included anti-BrdU (Abcam, Cambrige, UK), anti-laminin (Sigma-Aldrich), anti-vWF (Millipore, Billerica, MA, USA), anti-MMP-9 (Santa Cruz Biotechnology, CA, USA), anti-MMP-2 (Santa Cruz), anti-Ang-1 (Santa Cruz), anti-Tie-2 (Santa Cruz), anti-βIII-tubulin (Sigma-Aldrich), and anti-GFAP (Santa Cruz) antibodies. After incubation with CY2- or CY3-conjugated secondary antibodies (Invitrogen, San Diego, CA, USA) for 0.5 h, mounting medium with 4′-6-diamidino-2-phenylinidole (DAPI) (Zhong Shan Golden Bridge Biotechnology, Beijing, China) was used to label cell nuclei. The fluorescence signals were visualized using a microscope system (Nikon eclipse 80i, Japan) and a confocal laser scanning microscope system (MRC1024, Bio-Rad, Hercules, CA, USA).

For immunohistochemistry, the ice-cold brain sections were stained with primary antibodies, including anti-MMP-2 (Boster, Wuhan, Hubei, China), anti-MMP-9 (Santa Cruz), anti-CD34 (Abnova, Taipei, Taiwan), anti-Ang-1 (Boster), anti-Tie-2 (Santa Cruz) antibodies. The secondary antibodies were biotinylated horse anti-mouse and goat anti-rabbit IgG (Santa Cruz). The sections were stained using the avidin-biotin peroxidase complex method with 3,3′-diaminobenzidine as a chromogen, followed by being dehydrated in a graded alcohol series, cleared in xylene, and coverslipped with neutral balsam. A light microscope equipped with a computerized image analysis system (Olympus BX51, Japan) was used to examine the stained sections.

**Figure 1:**
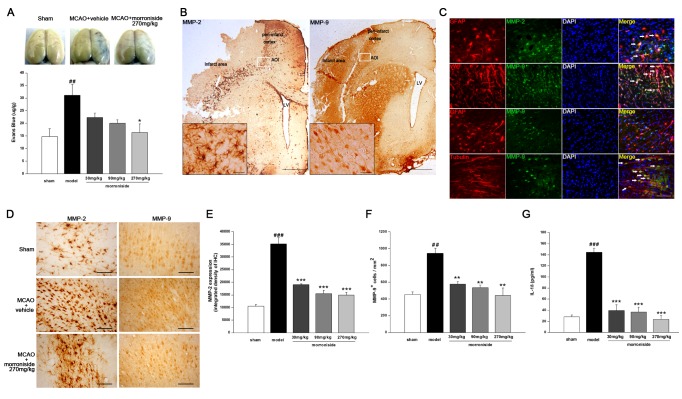
Morroniside regulates cerebrovascular permeability 72 h after cerebral ischemia. (A) Representative images of the brains from sham-operated rats, vehicle-treated ischemic rats and 270 mg/kg/day of morroniside-treated rats injected with Evans Blue. For quantitative analysis of EB levels, n = 5 for each group. (B) Immunohistochemistry of MMP-2 (left) and MMP-9 (right), represents the peri-infarct cortex and infarct area in the ischemic hemisphere with reperfusion of 72 h. White boxes indicated AOIs in the peri-infarct cortex. Scale bar, 1000 µm, and 25 µm for the enlarged images of MMP-2 or MMP-9. (C) Immunofluorescence micrographs show double-labeled GFAP and MMP-2 in ischemic hemispheres, indicating the expression of MMP-2 in astrocytes (arrows). Double-immunofluorecence staining of MMP-9 with vWF or βIII-tubulin indicates the predominate expression of MMP-9 in endothelial cells and neurons (arrows). Double immunostaining shows no colocalization between astrocytes and MMP-9. DAPI (blue) used to stain nuclei. DAPI (blue) was used to stain nuclei. Scale bar, 50 µm. (D) Sections from the brains of a sham-operated rat, vehicle-treated ischemic rat and 270 mg/kg/day of morroniside-treated rat stained with antibodies against MMP-2 or MMP-9 expressed in the peri-infarcted cortex. Scale bar, 100 µm. (E,F) Quantitative analysis of the density intensity of MMP-2 (E) and MMP-9-positive cells (F) (n = 3 for each group). (G) Quantitative analysis of IL-1β levels in the ipsilateral cortex as detected by ELISA (n = 6 for each group). The values are the mean ± SEM. ^##^ *p<*0.01, ^###^ *p<*0.001 versus sham-operated control group; *p<0.05, **p<0.01, ***p<0.001 versus vehicle-treated ischemic group.

All image analyses were conducted by observers who were blinded to the treatment conditions. Every 10th section between Bregma levels +1.60 and −0.2 mm were selected (total = 3 sections per brain). MMP-9^+^ cells, MMP-2^+^ cells and CD34^+^ vessels in the peri-infarct cortex were counted using a 40X objective (400 µm×300 µm) on a light microscope (Olympus BX51). Three areas of interest (AOIs) were selected around the margin of infarct area as described in Figure 1B, to obtain an average of the number of cells in peri-infarct cortex for each section. An average for the three slices per hemisphere was then taken. The digitalized images were contrast-enhanced to clearly differentiate positive staining from the background, and a thresholding procedure was established by using Image Pro Plus 5.0 software (Media Cybernetics, Inc., Bethesda, MD, USA) to determine the number of immunoreactive cells within each fixed field of view as previously described [18]. The counting of BrdU^+^/laminin^+^ cells in the peri-infarct area was performed using a confocal laser microscope of three sections per animal. AOIs were scanned with a ×40 objective lens in 260.6µm×260.6µm format in the x-y direction. For semi-quantitative measurement of vWF-labeled vessels, only the vWF^+^ vessels with a clear lumen and those vessels that formed ring-like or tubular structures were counted. Three sections per brain from the same levels were selected as described above. The number of vessels in each field (AOI = 560 µm×560 µm; three squares per section as described in the figure of vWF-immunostaining) was converted into the number of vessels/mm^2^. No correction was done for blood vessels that extended spatially over a sufficiently long distance.

### Protein expression analysis

After the rats were euthanized under 10% chloral hydrate (0.4 ml/kg) anesthesia, the whole cortex on the infarct side was harvested for an assay of proteins involved in the angiogenesis process and BBB permeability. The brain tissue was homogenized in ice-cold buffer (tris-(hydroxymethyl)-aminomethane 50 mM, pH 7.4, NaCl 150 mM, 0.5% Triton X-100, edetic acid 1 mM, phenylmethylsulfonyl fluoride 1 M, lepeptin 10 µg/ml and aprotinin 60 µg/ml) and centrifuged at 14,000×*g* at 4°C for 30 min to collect supernatants as the total protein. The proteins were then electrophoresed through a 10–15% sodium dodecyl sulfate polyacrylamide gel (SDS-PAGE) and electrically transferred onto a nitrocellulose membrane. This membrane was detected using primary antibodies, including anti-Ang-1(Boster), anti-Tie-2 (Santa Cruz), anti-neuropilin-1 (NRP-1) (Abcam), anti-fibroblast growth factor-2 (FGF-2) (Santa Cruz), and anti-vascular endothelial growth factor (VEGF) (Santa Cruz). The amount of protein was normalized with β-actin values in the same lane.

For the IL-1β content assay, an ELISA kit (R&D, Minneapolis, US) was applied. The protein supernatants were collected as described above and detected with IL-1β according to the manufacturer’s instructions.

### Statistical analysis

All data are presented as the mean ± SEM; *n* was the number of animals in each experiment. The statistical significance of the mean was calculated by analysis of variance (ANOVA) followed by Tukey’s multiple range post hoc test using Statistical Package for the Social Sciences software (SPSS 13.0 Statistical Software; SPSS Inc., Chicago, IL, USA). A value of *p*<0.05 was considered significant.

## Results

### Morroniside regulates cerebrovascular permeability 3 days after cerebral ischemia

In order to provide the direct evidence whether morroniside could protect the BBB function, Evans Blue dye was used to assess BBB integrity at 72 h after MCAO. The results indicated that Evans Blue extravasation was increased by approximately 1.1-fold in ischemic rats compared to the sham-operated group, indicating the disruption of BBB (Figure 1A). However, rats treated with morroniside at the dose of 270 mg·kg^−1^·day^−1^ showed a 43% reduction in Evans Blue extravasation after MCAO compared with the vehicle-treated ischemic rats (Figure 1A). There was also an decreasing tendency in 30 and 90 mg·kg^−1^·day^−1^ morroniside-treated animals compared with vehicle-treated ischemic rats, but no significant difference. The results suggested that morroniside could inhibit the disruption of cerebrovascular permeability during the acute phase after ischemic stroke.

### Morroniside alleviates MMPs activity and inflammatory reaction in BBB injury 3 days after cerebral ischemia

It has been shown that the activation of MMPs plays a crucial role in BBB injury after ischemic stroke [19], [20]. Excessive MMP activity can degrade the extracellular matrix and induce extensive blood vessel damage. We examined whether MMPs are involved in the mechanism of early BBB repair with treatment of morroniside. MMP-2 was demonstrated to be expressed in astrocytes around the margin of infarct areas by using double-immunofluorecence staining of MMP-2 with GFAP (Figure 1C). This is in accordance with the immunohistochemistry staining of MMP-2, which demonstrated astrocytic-like staining (Figure 1B). In contrast, MMP-9^+^/vWF^+^ cells and MMP-9^+^/βIII-tubulin^+^ cells were observed in the peri-infarct cortex, indicating MMP-9 was mainly expressed in endothelial cells and neurons 3 days post MCAO (Figure 1C). Immunohistochemistry staining of MMP-9 in the ischemic cortex showed obvious neuronal immunoreactivity (Figure 1B,D). As Figure 1D–F show, MMP-9 and MMP-2 levels were both increased in the peri-infarct cortex of ischemia-treated rats compared with sham-operated group at 72 h after ischemia-reperfusion, which is consistent with previous reports [21] (MMP-9^+^ cells·mm^−2^, MCAO, 941.9±61.5 versus sham, 452.19±31.78, *p*<0.01; integrated density of MMP-2, MCAO, 35160.7±2458.38 versus sham, 10550.49±653.22, *p*<0.001, n = 3). Morroniside could obviously reduceapp:addword:obviously the expression of MMP-2 (Figure 1E) and MMP-9 (Figure 1F) in the peri-infarct area compared with vehicle-treated ischemic rats, which gives some evidence that morroniside may protect BBB function after stroke via the suppression of MMPs.

BBB breakdown after stroke has also been associated with inflammation [22],[23]. In particular, excessive amounts of pro-inflammatory cytokines such as interleukin-1beta (IL-1β) can reduceapp:addword:reduce BBB function [24], [25]. So the expression of IL-1β was assessed at 72 h after MCAO by ELISA. The results indicated that levels of IL-1β were increased about 4.1-fold in ischemia-treated rats compared with the sham-operated group (Figure 1G; MCAO, 144.3±7.8 pg·ml^−1^ versus sham, 28.01±3.2 pg·ml^−1^, *p*<0.001; n = 6). At the concentration of morroniside 30 mg/kg, 90 mg/kg, 270 mg/kg respectively, it was found that morroniside could significantly reduce IL-1β content to control levels (Figure 1F; *p*<0.001, *p*<0.001, *p*<0.001, n = 6). Taken together, these results suggested that morroniside might protect the BBB partially by alleviating inflammatory reaction and BBB leakage.

### Morroniside promotes endothelial progenitor cells (EPCs) proliferation 7 days after cerebral ischemia

**Figure 2:**
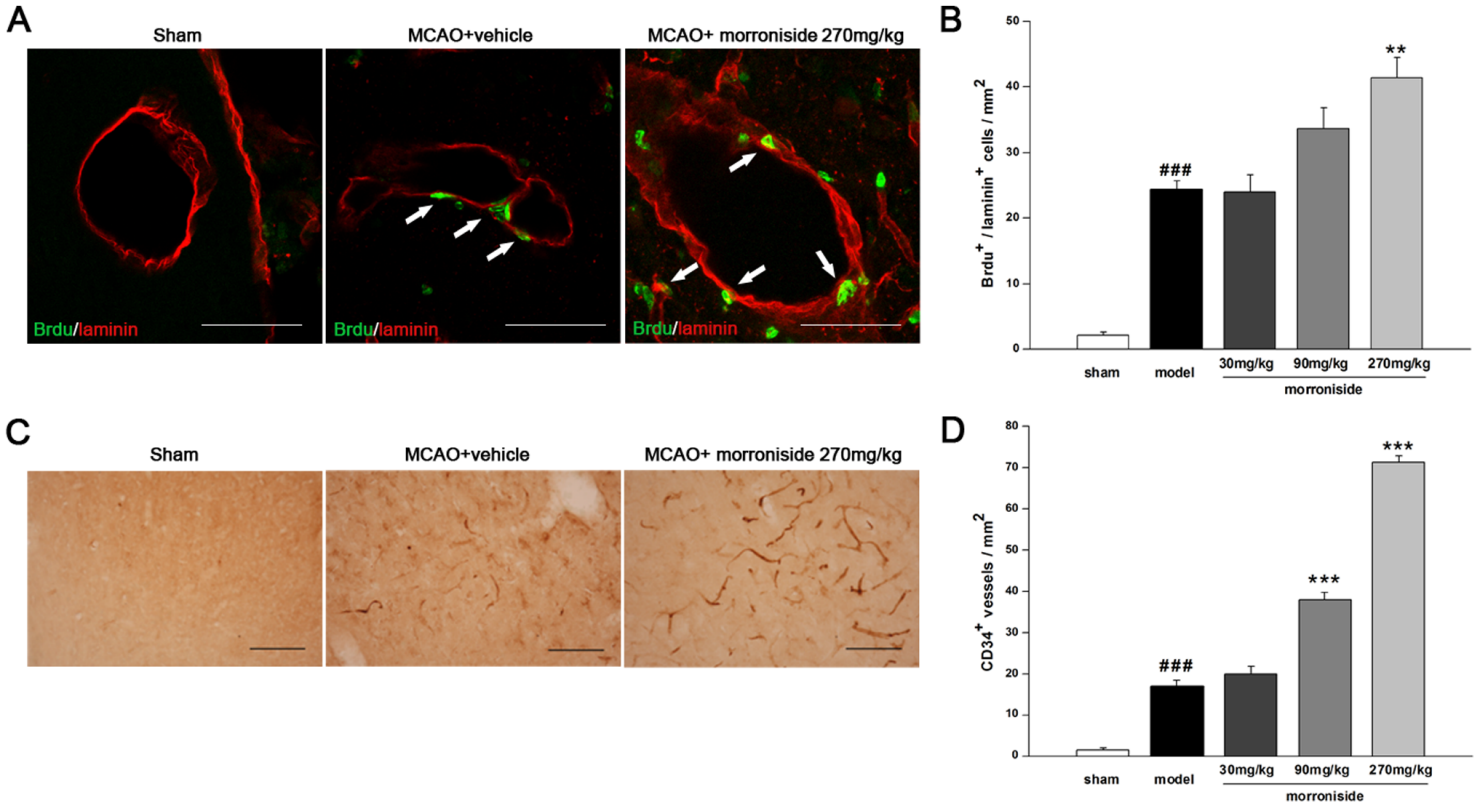
Morroniside promotes EPCs proliferation 7 days after cerebral ischemia. (A) Confocal microscopy images of newly born vascular endothelial cells labeled with BrdU^+^/laminin^+^ in the peri-infarcted cortex 7 days after MCAO (arrows). (B) Quantitative analysis of the number of BrdU^+^/laminin^+^ cells (n = 3 for each group). (C) Representative immunohistochemical images of CD34-positive vessels in the peri-infarcted cortex 7 days after MCAO. (D) Quantitative analysis of the number of CD34-positive vessels (n = 3 for each group). The values are the mean ± SEM. ^###^ *p<*0.001 versus sham-operated control group; **p<0.01 and ***p<0.001 versus vehicle-treated ischemic group. Scale bar, 50 µm.

To study whether morroniside could promote angiogenesis in the chronic microvessel integrity recovery, we next examined the effects of morroniside on the recruitment of EPCs in infarct brain areas 7 days after MCAO. Laminin is a basal membrane marker of blood vessels. Newborn vascular endothelial cells labeled with BrdU^+^/laminin^+^ were observed in the boundary of the ischemic cortex by means of the immunofluorescence, and the number of these double-positive cells was significantly increased about 71.1% with morroniside treatment at dose of 270 mg·kg^−1^·day^−1^ (Figure 2A,B; BrdU^+^/laminin^+^ cells·mm^−2^, morroniside, 41.35±3.06 versus MCAO, 24.24±1.47, *p*<0.01; n = 3). CD34 is an endothelial antigen that labels endothelial progenitor cells and could be used as an indirect marker of neoangiogenesis [26], [27]. Treatment with morroniside significantly increased the number of CD34^+^ vessels in the peri-infarct cortex (*p*<0.01) compared with the vehicle-treated ischemic group (Figure 2C,D). Taken together, these results suggested the beneficial effects of morroniside on EPCs proliferation in the peri-infarct region of the ischemic ipsilateral brain.

### Morroniside increases the expression of angiogenic promoters 7 days after cerebral ischemia

**Figure 3:**
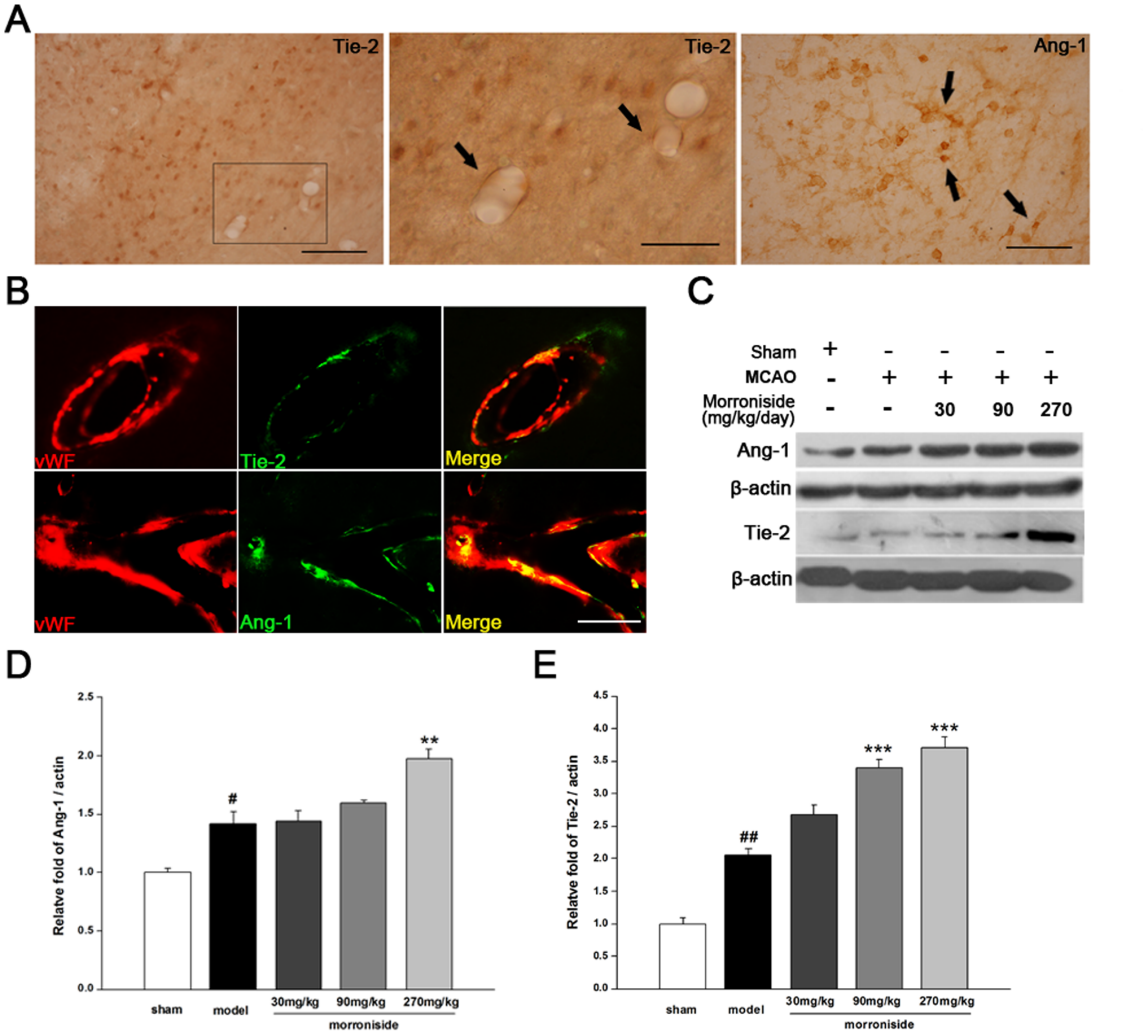
Effects of morroniside on the expression of Ang-1 and its receptor Tie-2 in the peri-infarct cortex of rats 7 days after cerebral ischemia. (A) Immunohistochemical staining of vessel-associated Tie-2 in the peri-infarct region (arrows), and new vessels with incomplete Ang-1-positive endothelial-like cell lining 7 days after ischemic/reperfusion treatment. Scale bar, 100 µm, and 50 µm for the enlarged image of Tie-2. (B) Double immunofluorecence staining of vWF with Ang-1 and Tie-2 indicated Ang-1 and Tie-2 were localized in endothelial cells. Scale bar, 50 µm. (C) Protein expression of Ang-1 (60 kDa) and Tie-2 (140 kDa) in the ipsilateral cortex 7 days after MCAO by immunoblotting. Quantitative analysis of Ang-1 and Tie-2 is expressed as a fraction of the respective levels of β-actin. n = 3 for each group. The values are the mean ± SEM. ^#^ *p<*0.05 and ^##^ *p<*0.01 versus sham-operated control group; **p<0.01 and ***p<0.001 versus vehicle-treated ischemic group.

**Figure 4:**
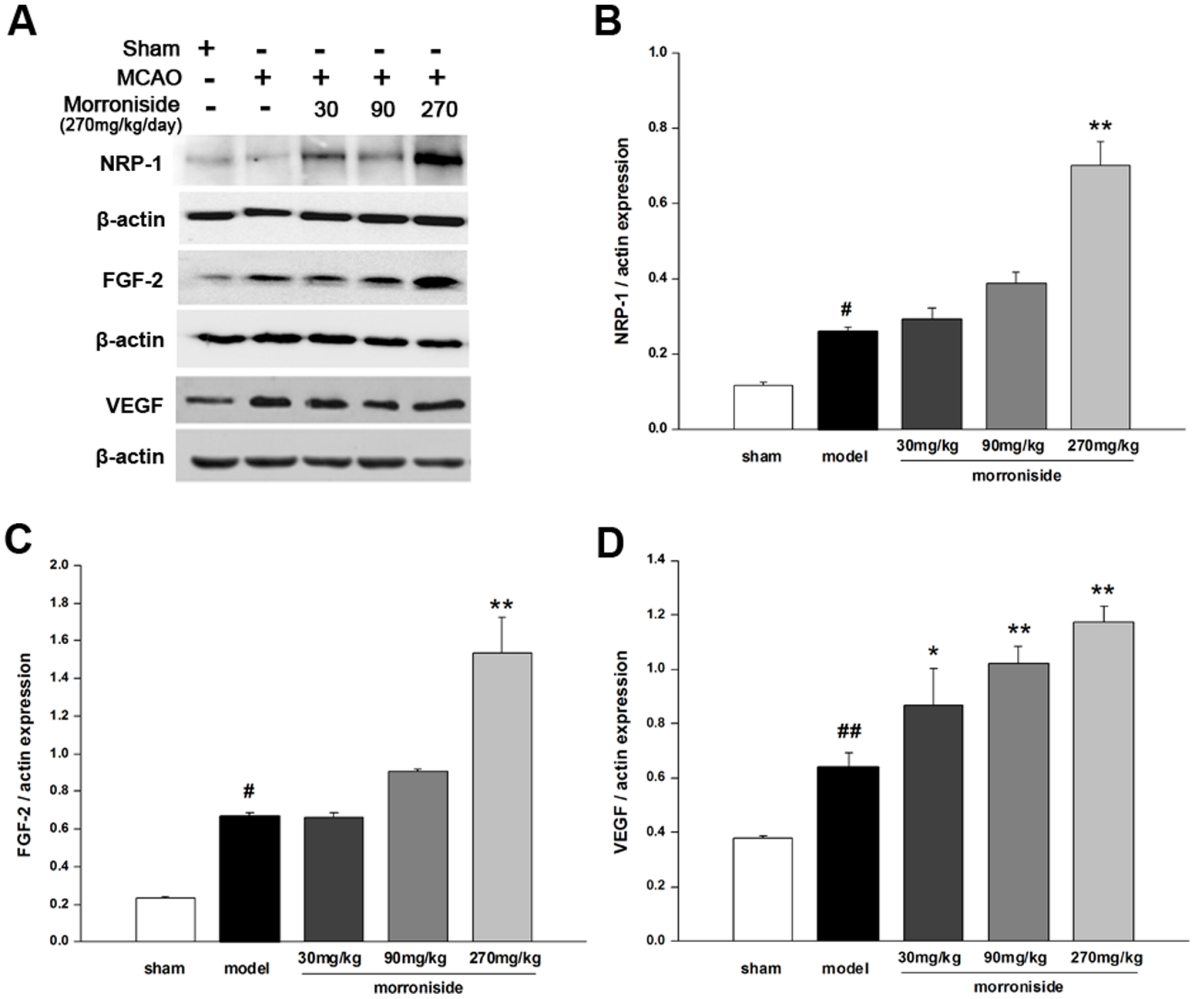
Effects of morroniside on the expression of angiogenic factors in the ischemic cortex of rats 7 days after cerebral ischemia. (A) Representative images of immunoblot analysis of NRP-1 (120 kDa), FGF-2 (20 kDa) and VEGF (25 kDa) in the ischemic ipsilateral cortex 7 days after MCAO. (B-D) Quantitative analysis of NRP-1 (B), FGF-2 (C) and VEGF (D) expressed as a fraction of the respective levels of β-actin. n = 3 for each group. The values are the mean ± SEM. ^#^ *p<*0.05 and ^##^ *p<*0.01 versus sham-operated control group; *p<0.05 and **p<0.01 versus vehicle-treated ischemic group.

Next, we examined the expression of angiogenic factors involved in the angiogenesis process 7 days after cerebral ischemia. In the remodeling peri-infarct cortex, new vessels showed incomplete Ang-1-positive endothelial-like signals along with vessel-associated Tie-2 expression (Figure 3A). Experiments of double immunostaining revealed that Tie-2 and Ang-1 were predominantly expressed in endothelial cells around the margin of infarct areas where active vessel remodeling was noted (Figure 3B). Treatment with morroniside increased the expression of Tie-2 and Ang-1 with similar effects observed by immunoblotting (Figure 3C–E; n = 3). We also examined the expression of NRP-1, FGF-2 and VEGF in the cortex of ischemic ipsilateral brain, and found the expression of NRP-1, FGF-2 and VEGF were increased by 7 days-ischemia compared with the sham-operated group (Figure 4A–D; *p*<0.05; *p*<0.05; *p*<0.01). Treatment with morroniside of 270 mg·kg^−1^·day^−1^ significantly increased the activation of NRP-1 and FGF-2 (Figure 4B,C; *p*<0.01; *p*<0.01). Moreover, treatment with morroniside at dose of 30, 90 and 270 mg·kg^−1^·day^−1^ upregulated the VEGF expression in a dose-dependent manner (Figure 4D). These results indicated that morroniside might promote angiogenesis after ischemic injury by activating the angiogenic signal and releasing the promoters.

### Morroniside increases the vWF^+^-vessels of ipsilateral peri-infarct cortex 7 days and 28 days after cerebral ischemia

**Figure 5:**
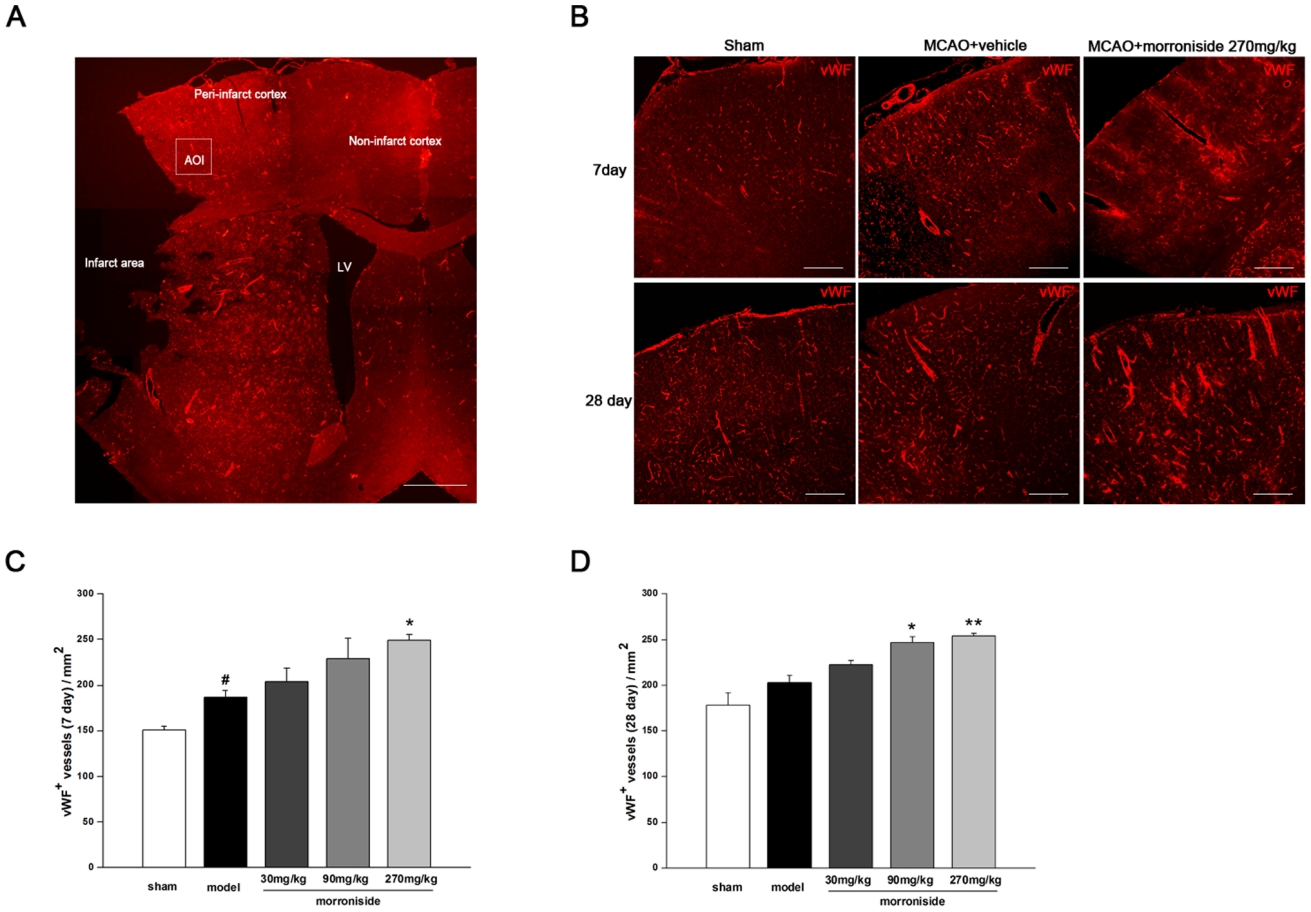
Morroniside increases the vWF^+^-vessels of ipsilateral peri-infarct cortex 7 days and 28 days after cerebral ischemia. (A) Photomontage of vWF-immunostaining represents microvessels in ischemic hemisphere 7 days after MCAO (nine micrographs were put together). The infarct area and peri-infarct area were indicated. A higher density of microvessels was seen in the peri-infarct area. White box indicated an AOI selected in the peri-infarct cortex. Scale bar, 1000 µm. (B) Representative microscopy images of vWF^+^ vessels in the peri-infarcted cortex of rats 7 days and 28 days after MCAO in sham-operated rats, a vehicle-treated ischemic group and 270 mg/kg/day of morroniside-treated rats. Scale bar, 250 µm. (C,D) Quantitative analysis of the number of vWF^+^ vessels 7 days (C) and 28 days (D) after ischemia. n = 3 for each group. The values are the mean ± SEM. *p<0.05 and **p<0.01 versus vehicle-treated ischemic group.

In order to obtain more evidence on promoting the recovery of vascular element within neurovascular by morroniside-treatment, immunofluorescence was applied to label the vessels with vWF. The results showed an increase in vWF^+^ vessels in the ipsilateral peri-infarct cortex 7 days after MCAO and an absence of vWF-immunoreactive vessels in the infarct core (Figure 5A). The number of vWF^+^ vessels was higher in the 270 mg·kg^−1^·day^−1^ morroniside-treated group than that in the vehicle-treated ischemic rats (Figure 5B,C; morroniside 270 mg·kg^−1^·day^−1^, 254.88±1.19 versus MCAO, 197.3±8.05, *p*<0.05; n = 3). Furthermore, vWF^+^ vessels were also detected 28 days after ischemia. The results showed that treatment with morroniside at the concentration of 90 and 270 mg·kg^−1^·day^−1^ could significantly sustain the increase in the amount of vessels in the penumbra, although there is no difference between the vehicle-treated ischemic rats and the sham-operated rats 28 days after ischemia (Figure 5B,D; vWF^+^ vessels·mm^−2^, MCAO, 202.7±7.75 versus sham, 178.53±13.42, *p*>0.05; morroniside 90 mg·kg^−1^·day^−1^, 246.43±6.47 versus MCAO, *p*<0.05; morroniside 270 mg·kg^−1^·day^−1^, 253.67±2.97 versus MCAO, *p*<0.01; n = 3). These results suggested that morroniside might enhance the revascularization of ischemic brain by promoting the elongation of new generated vessels.

## Discussion

Recovery from cerebral ischemia is a complex and highly dynamic process that includes both initial injury and response signals as well as the activation of endogenous self-preservation processes [28]. A key aspect of neurovascular unit protection after stroke involves preservation of BBB function [4], especially the microvascular integrity of neurovascular unit. In previous reports [14]–[16], we identified morroniside, a primary active ingredient extracted from the Chinese herb *C. officinalis*, as a potent acute neuroprotectant both in an oxidative stress-induced neurotoxic cell model and a focal cerebral ischemia rat model. Here, we focused on the role of morroniside in protecting and restoring the BBB integrity to better assess its effects on ischemic stroke recovery. Our findings suggested that morroniside could inhibit the acute injury on microvascular integrity and could promote angiogenesis for neurovascular recovery after stroke.

Ischemia–reperfusion in the brain triggers oxidative and nitrative injury in the neurovascular unit [29]. High levels of superoxide and peroxynitrate production observed in neurons, astrocytes and the endothelium, are associated with vascular injury and blood–brain barrier breakdown [30]. In the present study, the leakage of Evans Blue indicated BBB breakdown 3 days post-ischemia. Morroniside inhibited the disruption of cerebrovascular permeability, suggesting the protective effects on acute BBB injury. Expression of MMPs has been shown to be significantly increased during stroke in human and rat models of focal ischemia [31]–[33]. MMP-9 is activated within 24 h to months after ischemic episode, while MMP-2 is increased by 2–5 days because its activation requires the presence of TIMP-2 [21]. Consistent with these data, we showed that MMPs were upregulated on 3 days post-injury. This increase in the level of MMPs contributed to the disruption of the BBB, leading to vasogenic cerebral edema in acute ischemia. Besides, we showed that MMP-2 was expressed by astrocytic endfeet 3 days after MCAO, and MMP-9 was localized within endothelial cells and neurons, which is consistent with previous studies [13], [34]. It is also thought that focal ischemia of the brain is a pro-inflammatory stimulus and that interactions of inflammatory cytokines with components of the neurovasculature are in response to BBB permeability [23]. Our studies supported this hypothesis and identified IL-1β as a stimulus of endothelial permeability, which has been previously postulated to facilitate edema formation during the initial moments following the onset of focal ischemia [35], [36]. On the basis of these results, we conjectured that morroniside regulated cerebrovascular permeability after stroke via the downregulation of MMPs and IL-1β, hence inhibiting edema formation during the initial phase. However, the effects of morroniside on MMPs are different from those of MMP inhibitors which are used to restore the early integrity of the BBB in rodent ischemia models but are ineffective in the later opening at 48 h [37]. Direct MMP inhibitors could block or slow down neurorepair from stroke, since MMPs are involved in both angiogenic and neurogenic processes. However, successive administration of morroniside for 7 days after ischemia-injury significantly promoted angiogenesis process to restore microvascular functional integrity.

Angiogenesis is important for chronicapp:addword:chronic stroke recovery, since angiogenic stimulation generates new vessels, which could increase the collateral circulation, repair the BBB functionality and provide the important scaffolds for NPC migration toward the damaged brain region. Researches on molecular mechanisms of post-ischemic angiogenesis have shown that angiogenesis is a multistep process including several developmental milestones, and most important, endothelial cell activation contributes to vascular homeostasis and repair mechanisms [38]–[40]. Our results showed that newborn vascular endothelial cells labeled with BrdU^+^/laminin^+^ were localized in the boundary of the ischemic cortex. Moreover, treatment with morroniside for 7 days after the ischemia significantly increased the number of newly formed vessels in the peri-infarct areas, as labeled with EPC and matured endothelial cell marker. At 28 days post ischemia, the number of vWF^+^ vessels in the penumbra of vehicle-treated ischemic rats was not significantly different from that in sham-operated rats. However, administration of morroniside (90 mg·kg^−1^·and 270 mg·kg^−1^) enhanced the amount of vWF^+^ vessels at 28 days after ischemia, suggesting that morroniside plays a crucial role in angiogenesis during the chronic phase.

Angiogenesis is demonstrated to be regulated by angiogenic growth factors, especially the VEGF/VEGF receptor (VEGFR) and the Ang-1/Tie-2 system [41]. We evaluated the expression of angiogenic growth factors involved to confirm the beneficial effects of morroniside on angiogenesis. As the most important and best-characterized angiopoietin, Ang-1 promotes endothelial cell survival and blood vessel maturation via binding to its receptor Tie-2, which is highly expressed in developing vasculature but is down-regulated in the vasculature of the adult brain [42], [43]. As reported in previous studies, significant increases in both protein levels and mRNA levels of Ang-1 and Tie-2 in the ischemic cerebral cortex was induced by transient ischemia, and this regulation may have an essential role in large vessel remodeling, maintenance of vascular structures, vasculogenesis, and nonsprouting angiogenesis [44], [45]. Consistent with these results, we showed that the expression of Ang-1 and Tie-2 were significantly increased in the ischemic cerebral cortex by treatment with morroniside. VEGF is the most important mitogen in the process of angiogenesis, including microvascular permeability, endothelial cell proliferation, invasion, migration and survival [40], [46]. An increase in VEGF expression in the infarcted hemisphere has been described as early as 3 h after an ischemic insult and continued up to 7 days [47]. Administration of VEGF augmented angiogenesis in the ischemic penumbra and improve neurological recovery [48], [49]. FGF-2, one of the first discovered angiogenic factors, mediates vessel growth by stimulating the release of VEGF and other signaling, and is required for the maintenance of vascular integrity [50]. NRP-1, expressed in neurons, vessels and astrocytes, also has angiogenic properties, identified as co-receptors for VEGF or to form complexes with VEGFR [51]. It has also been reported to be increased by FGF-2 to enhance vascular smooth muscle cell migration, besides guidance of endothelial tip cell [52]. After ischemic insult, NRP-1 was upregulated in endothelial cells of cerebral blood vessels at the border and in the core of the ischemic lesion 7 days [53], [54]. The results showed that the increased expression of VEGF, FGF-2, and NRP-1 induced by ischemia were enhanced after administration of morroniside for 7 days, indicating that morroniside might promote angiogenesis by activating the angiogenic signal and releasing the promoters. Further studies will be needed to investigate how morroniside interacts with these proteins and the impact of these interactions on angiogenesis after ischemic injury.

**Figure 6:**
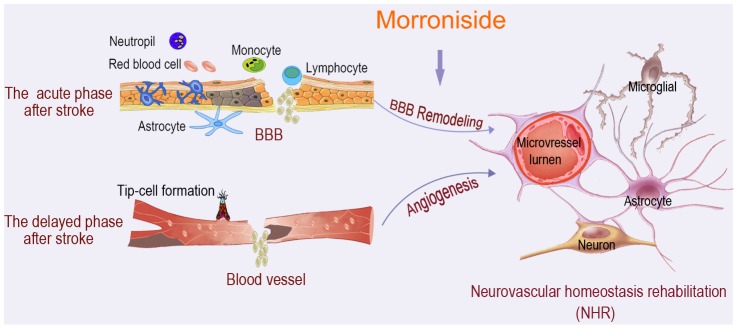
Schematic diagram of morroniside on neurovascular homeostasis rehabilitation (NHR) hypothesis. This diagram presents a new model proposed for restoration of microvessel integrity of neurovascular unit following ischemia. Cerebral ischemia induces not only initial injury on the vascular compartments of the neurovascular unit, but also the activation of endogenous self-preservation processes during the delayed phase. However, morroniside-treatment could regulate BBB permeability during the initial phase following the onset of focal ischemia and could facilitate brain self-repair processes of angiogenesis, finally promoting blood-brain barrier remodeling in response to ischemia. It suggests a potential strategy that targeting the vascular compartments in damaged brain by using exogenous drugs for neurovascular homeostasis rehabilitation.

On the basis of these initial results, we concluded that morroniside could regulate BBB permeability by downregulation of MMPs and IL-1β during the initial phase following the onset of focal ischemia; and could promote angiogenesis for BBB remodeling by activation of EPCs proliferation and increase of angiogenic promoters expression during the delayed phase. Accordingly, we propose a new model of the restoration of microvascular integrity in the neurovascular unit and suggest a potential treatment strategy that targets the vascular components in the damaged brain by use of exogenous drugs such as morroniside to restore neurovascular homeostasis (Figure 6). Our data suggest that (i) reconstruction of BBB function is necessary for brain repair after ischemic stroke, and (ii) stroke recovery therapy should be oriented toward stimulating and prolonging endogenous neurorepair processes, including neovascularization. One caveat remains, i.e. the temporal relationship between acute neuroprotection and delayed neurorepair. Treatments with morroniside in this intial study were started at 3 hours after MCAO. Thus, overlap between its known acute protective effects and our present findings of promoted angiogenesis in the delayed phase after stroke cannot be fully separated, and ultimately, the correlation between delayed neurorepair effect and self-repair ability still needs to be fully defined. Further studies to define the exact mechanisms underlying angiogenic recovery and the vascular signals and substrates involved in the neurovascular repair are warranted.
